# Short-term and four-year feeding and respiratory outcomes of infants with micrognathia

**DOI:** 10.1038/s41372-025-02224-1

**Published:** 2025-04-12

**Authors:** Kuan-Chi Lai, Laura M. Walker, Kevin Moran, Jordan W. Swanson, Jesse A. Taylor, Janet Lioy, Christopher M. Cielo

**Affiliations:** 1 Division of Neonatology, The Children’s Hospital of Philadelphia, Philadelphia, PA USA; 2 Division of Neonatology, Children’s Hospital Los Angeles, Los Angeles, CA USA; 3 Department of Pediatrics, The Children’s Hospital of Philadelphia, Philadelphia, PA USA; 4 Division of Plastic, Reconstructive and Oral Surgery, The Children’s Hospital of Philadelphia, Philadelphia, PA USA; 5 Division of Pulmonary and Sleep Medicine, The Children’s Hospital of Philadelphia, Philadelphia, PA USA

**Keywords:** Paediatrics, Outcomes research, Risk factors

## Abstract

**Objective:**

To describe feeding and respiratory outcomes at discharge and at the most recent follow-up visit prior to four years old in infants evaluated for micrognathia.

**Study design:**

Single-center retrospective analysis of 218 patients admitted and evaluated with congenital micrognathia during infancy. Outcomes were compared based on treatment (medical, mandibular distraction osteogenesis, or tracheostomy), and also compared based on syndromic status.

**Results:**

Tube feeding was required by 81% of infants at discharge and 41% at follow-up. Respiratory support was required by 32% at discharge and 22% at follow-up. There were no differences in feeding and respiratory support at discharge and at follow-up between medical treatment and mandibular distraction osteogenesis. Tracheostomy was associated with more tube feeding and respiratory support at both discharge and at follow-up. Genetic syndromes were more likely to require tube feeding and respiratory support.

**Conclusion:**

Long-term feeding and respiratory support are common in infants hospitalized with micrognathia.

## Introduction

Micrognathia is a common congenital anomaly occurring in as many as 1:1000 to 1:1600 births [1, 2]. Congenital micrognathia may present as an isolated finding, part of the Robin sequence with or without cleft palate, or as part of a more complex genetic syndrome, which may have implications for treatment options and prognosis [3, 4]. In the past decade, mandibular distraction osteogenesis (MDO) has replaced tongue-lip adhesion to become the most common surgical option for those with significant airway obstruction and may otherwise require tracheostomy [4–6]. However, there is currently no standardized guideline to inform clinicians the ideal candidacy for MDO, optimal timing of surgery, perioperative management, or the overall care of infants with micrognathia. Therefore, understanding the expected short-term and long-term outcomes can be important for family counseling and decision-making by the multi-disciplinary team caring for infants with micrognathia.

Several studies have reported outcomes of infants with micrognathia. A substantial proportion of hospitalized infants with micrognathia require tube feeding, and infants with underlying genetic syndromes or those needing surgical intervention are more likely to require gastrostomy tube [6–13]. MDO has high success rate (>95%) in avoiding tracheostomy [4, 6] and significantly improves obstructive sleep apnea [12, 14–16]. However, the majority of studies evaluating feeding and respiratory outcomes in infants with micrognathia were limited by small cohorts, included only those who underwent surgery, and/or lacked long-term follow-up after discharge from initial hospitalization.

The objective of this study is to describe the feeding and respiratory status at admission, discharge, and at follow-up visit up to four years of age. Additional outcomes including neonatal hospital length of stay (LOS), polysomnography findings, mortality, and other procedures are also evaluated. Outcomes are compared based on treatment type (medical treatment, MDO, tracheostomy), and also compared among syndromic status.

## Materials/subjects and methods

This was a single-center, retrospective cohort study of infants who were admitted to The Children’s Hospital of Philadelphia (CHOP) for the evaluation and management of micrognathia between 1/1/2010 and 2/28/2019. All infants who were admitted to the hospital and evaluated by plastic surgery for micrognathia prior to one year of age were eligible except for those who underwent prior MDO or tracheostomy at outside hospital prior to admission at our center. Patients were admitted from our Special Delivery Unit (inborn), emergency department, directly from clinic, or transferred from outside hospital. Patient outcomes were followed up to four years of age or until lost to follow-up. Authors LW and KM extracted data from the electronic health record and input into REDCap (Research Electronic Data Capture) for data collection and management [17]. Author KL then independently reviewed and verified data accuracy.

The evaluation and management of micrognathia at CHOP includes multi-disciplinary input from neonatology, plastic surgery, pulmonology, otolaryngology, and genetics, following a standardized protocol formalized circa 2017 and the management of patients prior to 2017 followed similar conceptual framework [18]. Generally, at our center, infants with micrognathia who have tongue-based obstruction and moderate/severe obstructive sleep apnea (OSA) and absence of severe genetic syndromes are considered favorable MDO candidates whereas those with severe genetic syndromes, poor neurocognitive prognosis, or multi-level airway obstructions are unfavorable MDO candidates and tracheostomy is preferred [18, 19]. Patients with less severe OSA are generally managed non-surgically with prone positioning, continuous positive airway pressure or supplemental oxygen during sleep with close multidisciplinary follow-up and re-evaluation.

Patient demographics and clinical characteristics including gestational age, birthweight, sex, race/ethnicity, syndromic status, intubation status at birth, and source of admission were directly extracted from the electronic health record. In this study, treatment group assignment was based on the initial surgical treatment of MDO (*n* = 119) or tracheostomy (*n* = 25), and those without either MDO or tracheostomy were assigned as medical treatment (*n* = 74). Syndromic status was characterized by confirmed syndromic (with positive genetic tests), suspected syndromic (patients with clinical features suspected to be related to a syndrome but with negative or unknown genetic tests), and non-syndromic (including isolated Robin sequence). Feeding route (exclusively by mouth, nasogastric tube, gastrostomy tube, nothing by mouth) and respiratory status (room air without respiratory support, standard nasal cannula or high-flow nasal cannula, continuous positive airway pressure or non-invasive mechanical ventilation, invasive ventilation, tracheostomy) at admission, at discharge, and at last follow-up were determined from chart review. Weight trajectory at discharge was assessed using the difference in weight Z-score at discharge from the birthweight Z-score. Obstructive apnea-hypopnea index (OAHI) and oxyhemoglobin saturation (SpO2) nadir from polysomnography were extracted for analysis.

Categorical variables were summarized by frequencies and percentages, and compared by the Fisher’s exact test. Continuous variables were summarized by medians and interquartile ranges, and compared by the non-parametric Wilcoxon rank-sum test. The comparison of polysomnography data before and after MDO was conducted by the non-parametric Wilcoxon signed-rank test for paired samples. Multivariable logistic regression models adjusting for gestational age, birthweight, sex, race/ethnicity, and intubation status at birth, were used to evaluate the association of treatment group and syndromic status with feeding and respiratory status at discharge and at follow-up. Bonferroni correction applied to a significance level of 0.05 was used in each group of comparisons (patient characteristics, MDO characteristics, polysomnography, feeding and respiratory support, and other outcomes, separated by treatment group and syndromic status) to determine statistical significance (see each table for details). All analyses were performed by using SAS software, version 9.4 (SAS Institute Inc., Cary, NC).

## Results

**Fig. 1:**
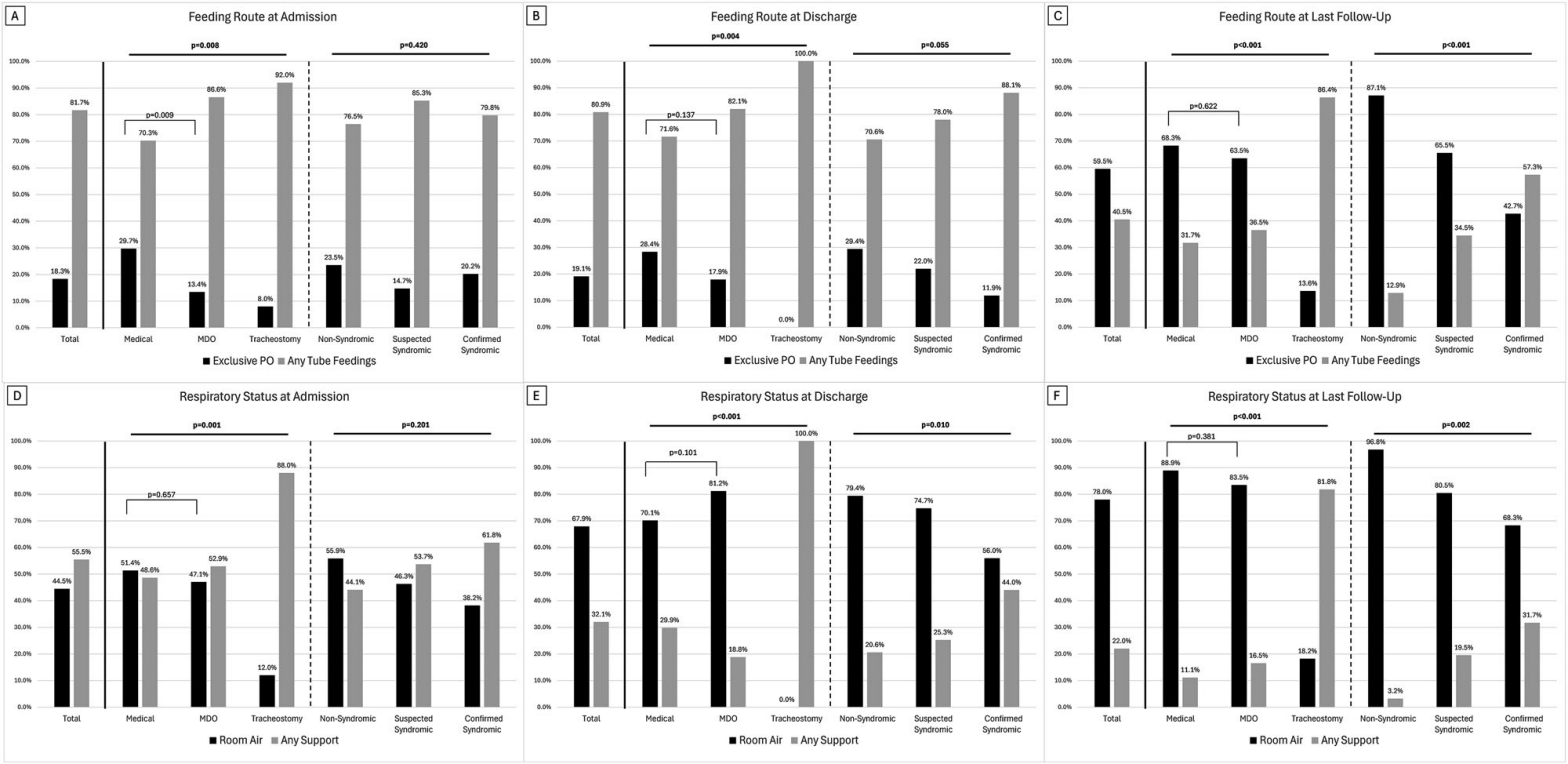
Feeding and Respiratory Outcomes by Treatment Group and Syndromic Status. Feeding route (**A**) at admission, **B** at discharge, and **C** at follow-up. Respiratory support (**D**) at admission, **E** at discharge, and **F** at follow-up.

A total of 218 infants with micrognathia, including 119 (55%) who underwent MDO and 25 (12%) who underwent tracheostomy as the initial surgical treatment, were admitted to CHOP between 1/1/2010 and 2/28/2019. Table 1 describes the patient characteristics. The majority of the cohort were full-term, male, non-Hispanic white, suspected or confirmed syndromic, and not intubated at birth. There were 34 (16%) patients who were non-syndromic, 95 (44%) patients who were suspected syndromic (with negative/unknown genetic tests), and 89 (41%) who were confirmed syndromic with positive genetic tests. The majority of suspected or confirmed genetic syndromes were Stickler syndrome (41/184, 22%), followed by other chromosomal duplication/deletion syndromes (17/184, 9.2%), and then 22q11.2 deletion syndrome (11/184, 6.0%). Supplemental Table 1 shows the full list of genetic syndromes. The majority of patients were evaluated from birth hospitalization either from the Special Delivery Unit (37, 17%) or from outside hospital (135, 62%), while the remaining were readmitted from clinic (24, 11%) or emergency department (22, 10%). The median (interquartile range) age at admission was 4 (0, 24) days for the overall cohort. Patients admitted from the Special Delivery Unit were admitted earlier (as they are inborn), followed by outside hospital, emergency department, and clinic: 0 (0, 0) days vs. 4 (1, 16) days vs. 31 (13, 58) days vs. 40 (14, 105) days, *p* < 0.001. Patients with confirmed or suspected syndromes were admitted earlier than non-syndromic patients: 2 (0, 10) days vs. 5 (1, 25) days vs. 14 (5, 39) days, *p* = 0.004. Infants with micrognathia who underwent medical treatment were more likely to exclusively feed by mouth at admission (30%) compared to infants who would go on to have MDO (13%) or tracheostomy (8%), *p* = 0.008 (Table 1 and Fig. 1A). A larger proportion of patients who underwent medical treatment (51%) or MDO (47%) were in room air at admission compared to those received tracheostomy later (12%), *p* = 0.001 (Table 1 and Fig. 1D). There were no significant differences in the proportion of patients able to exclusively feed by mouth or in room air at admission based on syndromic status.

**Table 1:** Patient characteristics.

	Total	Treatment Group^a^				Syndromic Status			
*N* (%) or Median (IQR)		Medical	MDO	Tracheostomy	*p*-value	Non-Syndromic	Suspected Syndromic	Confirmed Syndromic	*p*-value
**Total**	218 (100.0%)	74 (33.9%)	119 (54.6%)	25 (11.5%)		34 (15.6%)	95 (43.6%)	89 (40.8%)	
Treatment Group^a^					n/a				0.312
Medical	74 (33.9%)	n/a	n/a	n/a		12 (35.3%)	31 (32.6%)	31 (34.8%)	
MDO	119 (54.6%)	n/a	n/a	n/a		20 (58.8%)	56 (58.9%)	43 (48.3%)	
Tracheostomy	25 (11.5%)	n/a	n/a	n/a		2 (5.9%)	8 (8.4%)	15 (16.9%)	
Gestational Age (weeks)	38 (37–39)	39 (37–40)	38 (37–39)	37 (36–39)	0.025	38 (36–40)	39 (37–40)	38 (37–39)	0.779
Birthweight (grams)	2913 (2340–3400)	2915 (2354–3450)	2920 (2340–3319)	2810 (2330–3400)	0.697	3119 (2740–3544)	2954 (2380–3450)	2715 (2280–3280)	0.109
Sex					0.885				0.523
Female	88 (40.4%)	31 (41.9%)	48 (40.3%)	9 (36.0%)		13 (38.2%)	35 (36.8%)	40 (44.9%)	
Male	130 (59.6%)	43 (58.1%)	71 (59.7%)	16 (64.0%)		21 (61.8%)	60 (63.2%)	49 (55.1%)	
Race/Ethnicity					0.390				0.257
Non-Hispanic White	127 (58.3%)	43 (58.1%)	69 (58.0%)	15 (60.0%)		21 (61.8%)	55 (57.9%)	51 (57.3%)	
Non-Hispanic Black	22 (10.1%)	8 (10.8%)	13 (10.9%)	1 (4.0%)		1 (2.9%)	8 (8.4%)	13 (14.6%)	
Hispanic	34 (15.6%)	7 (9.5%)	21 (17.6%)	6 (24.0%)		3 (8.8%)	17 (17.9%)	14 (15.7%)	
Other/Unknown	35 (16.1%)	16 (21.6%)	16 (13.4%)	3 (12.0%)		9 (26.5%)	15 (15.8%)	11 (12.4%)	
Syndromic					0.312				n/a
Non-Syndromic	34 (15.6%)	12 (16.2%)	20 (16.8%)	2 (8.0%)		n/a	n/a	n/a	
Suspected Syndromic	95 (43.6%)	31 (41.9%)	56 (47.1%)	8 (32.0%)		n/a	n/a	n/a	
Confirmed Syndromic	89 (40.8%)	31 (41.9%)	43 (36.1%)	15 (60.0%)		n/a	n/a	n/a	
Intubation Status at Birth					0.005				0.749
No	152 (69.7%)	58 (78.4%)	81 (68.1%)	13 (52.0%)		24 (70.6%)	69 (72.6%)	59 (66.3%)	
Yes	52 (23.9%)	9 (12.2%)	31 (26.1%)	12 (48.0%)		7 (20.6%)	20 (21.1%)	25 (28.1%)	
Unknown	14 (6.4%)	7 (9.5%)	7 (5.9%)	0 (0.0%)		3 (8.8%)	6 (6.3%)	5 (5.6%)	
Age at Initial Admission (days)	4 (0–24)	5 (1–22)	5 (1–33)	1 (0–6)	0.077	14 (5–39)	5 (1–25)	2 (0–10)	0.004
Source of Admission					0.002				0.012
Clinic	24 (11.0%)	6 (8.1%)	18 (15.1%)	0 (0.0%)		3 (8.8%)	15 (15.8%)	6 (6.7%)	
Emergency Department	22 (10.1%)	14 (18.9%)	7 (5.9%)	1 (4.0%)		6 (17.6%)	10 (10.5%)	6 (6.7%)	
Outside Hospital	135 (61.9%)	43 (58.1%)	78 (65.5%)	14 (56.0%)		24 (70.6%)	57 (60.0%)	54 (60.7%)	
Special Delivery Unit^b^	37 (17.0%)	11 (14.9%)	16 (13.4%)	10 (40.0%)		1 (2.9%)	13 (13.7%)	23 (25.8%)	
Weight at Admission (grams)	3030 (2510–3580)	3188 (2640–3800)	2955 (2530–3550)	2820 (2330–3445)	0.109	3433 (2900–3800)	3025 (2530–3570)	2865 (2370–3455)	0.022
Feeding Route at Admission					0.008^e^				0.420^e^
Exclusively by Mouth	40 (18.3%)	22 (29.7%)	16 (13.4%)	2 (8.0%)		8 (23.5%)	14 (14.7%)	18 (20.2%)	
Partial Nasogastric Tube Feedings	22 (10.1%)	6 (8.1%)	16 (13.4%)	0 (0.0%)		7 (20.6%)	10 (10.5%)	5 (5.6%)	
Nasogastric Tube Feeding Only	63 (28.9%)	16 (21.6%)	42 (35.3%)	5 (20.0%)		10 (29.4%)	31 (32.6%)	22 (24.7%)	
Gastrostomy Tube Feeding	10 (4.6%)	2 (2.7%)	6 (5.0%)	2 (8.0%)		1 (2.9%)	2 (2.1%)	7 (7.9%)	
Nothing By Mouth	83 (38.1%)	28 (37.8%)	39 (32.8%)	16 (64.0%)		8 (23.5%)	38 (40.0%)	37 (41.6%)	
Respiratory Status at Admission					0.001^f^				0.201^f^
Room Air	97 (44.5%)	38 (51.4%)	56 (47.1%)	3 (12.0%)		19 (55.9%)	44 (46.3%)	34 (38.2%)	
NC/HFNC^c^	40 (18.3%)	16 (21.6%)	22 (18.5%)	2 (8.0%)		7 (20.6%)	18 (18.9%)	15 (16.9%)	
CPAP/NIMV^d^	27 (12.4%)	5 (6.8%)	15 (12.6%)	7 (28.0%)		1 (2.9%)	10 (10.5%)	16 (18.0%)	
Invasive Ventilation	50 (22.9%)	15 (20.3%)	26 (21.8%)	9 (36.0%)		5 (14.7%)	23 (24.2%)	22 (24.7%)	
Tracheostomy	4 (1.8%)	0 (0.0%)	0 (0.0%)	4 (16.0%)		2 (5.9%)	0 (0.0%)	2 (2.2%)	

^a^The categorization of MDO and tracheostomy is based on the initial surgical treatment.

^b^Special Delivery Unit is the delivery unit within The Children’s Hospital of Philadelphia for the delivery of neonates with prenatally diagnosed congenital defects.

^c^Standard nasal cannula or high-flow nasal cannula.

^d^Continuous positive airway pressure or non-invasive mechanical ventilation.

^e^Comparing feeding exclusively by mouth vs. all kinds of tube feedings.

^f^Comparing room air vs. all kinds of respiratory support.

# Bonferroni correction applied (*p* = 0.05/9 = 0.0056) to patient characteristics separately for treatment group and syndromic status.

## Bonferroni correction for feeding route and respiratoy status at admission, discharge, and follow-up were grouped together (*p* = 0.05/6 = 0.0083, separately for treatment group and syndromic status).

Clinical characteristics specific to MDO are described in Table 2. Age at MDO as the initial surgical treatment was 37 (17, 70) days and time from admission to surgery for the 119 patients who underwent MDO as the initial treatment was 13 (7, 25) days. Non-syndromic patients had a tendency of earlier attempt of extubation after MDO: 5 (3, 6) days compared to 7 (5, 7) days for patients with suspected syndromes and 7 (5, 8) days for patients with confirmed syndromes, *p* = 0.008 (however not significant after Bonferroni’s correction). There were no failed attempts from the initial extubation for non-syndromic patients; however, 7.1% of patients with suspected syndromes and 26% of patients with confirmed syndromes failed the initial extubation attempt, *p* = 0.004. A total of 8 (6.7%) patients who failed extubation subsequently had tracheostomy and another 2 (1.7%) patients who were not able to extubate received tracheostomy.

**Table 2:** Clinical characteristics related to MDO.

	Total	Syndromic Status			
*N* (%) or Median (IQR)		Non-Syndromic	Suspected Syndromic	Confirmed Syndromic	*p*-value
**Total**	119 (100.0%)	20 (16.8%)	56 (47.1%)	43 (36.1%)	
Age at MDO (days)	37 (17–70)	37 (14–50)	40 (18–73.5)	34 (17–80)	0.522
Days from Admission to MDO	13 (7–25)	10 (7.5–13.5)	13 (7.5–25)	16 (7–32)	0.251
Weight at MDO (grams)	3500 (3055–4090)	3690 (3347–4238)	3515 (3070–3978)	3350 (2860–4130)	0.309
Days to Extubation Attempt after MDO	6.5 (5–7)	5 (3–6)	7 (5–7)	7 (5–8)	0.008
Failed Initial Extubation	15 (12.6%)	0 (0.0%)	4 (7.1%)	11 (25.6%)	0.004
Did Not Extubate (Tracheostomy)	2 (1.7%)	0 (0.0%)	0 (0.0%)	2 (4.7%)	0.278
Did Not Extubate (Expired)	1 (0.8%)	0 (0.0%)	0 (0.0%)	1 (2.3%)	0.529
Tracheostomy after Extubation Attempt	8 (6.7%)	1 (5.0%)	2 (3.6%)	5 (11.6%)	0.342
Days to Distractor Removal	89 (77–100)	84 (76.5–102)	89 (77–98)	91 (75–103)	0.939
Repeat MDO	10 (8.4%)	1 (5.0%)	6 (10.7%)	3 (7.0%)	0.746

# Bonferroni correction applied (*p* = 0.05/10 = 0.005).

Polysomnography was conducted during neonatal hospitalization in 67% of patients who did not undergo MDO and also pre-operatively in 77% of patients who underwent MDO (Supplemental Table 2). Patients who underwent MDO had a significantly higher OAHI than patients who did not undergo MDO: 45.7 (18.8, 130.1) events/hour vs. 18.3 (7.5, 36.6) events/hour, (*p* < 0.001) and lower SpO2 nadir: 79% (63%, 85%) vs. 84% (79%, 89%), (*p* = 0.005). Of the 95 patients who underwent MDO and had a pre-operative diagnostic polysomnography, 74 (78%) also were evaluated with a post-MDO polysomnography. Post-MDO polysomnography showed significant decrease in OAHI: 4.6 (1.5, 11.7) events/hour vs. 45.7 (18.8, 130.1) events/hour pre-operatively, (*p* < 0.001) and increase in SpO2 nadir (84% [81%, 90%] vs. 79% [63%, 85%], *p* = 0.013).

Other short-term outcomes at discharge from the initial hospitalization are shown in Table 3. LOS was significantly longer in patients who underwent tracheostomy or MDO vs. those with medical treatment only: 129 (67, 178) vs. 39 (29, 81) vs. 17 (9, 29) days, (*p* < 0.001). Infants with micrognathia had suboptimal weight gain at discharge. Z-score difference between weight at discharge and birthweight for the overall cohort was (−1.05 [−1.57, −0.67]). 43% of the overall cohort failed hearing test at discharge with those underwent tracheostomy (76%) or those with confirmed syndromes (56%) having higher likelihood of failing hearing test.

**Table 3:** Outcomes.

	Total	Treatment Group^a^				Syndromic Status			
*N* (%) or Median (IQR)		Medical	MDO	Tracheostomy	*p*-value	Non-Syndromic	Suspected Syndromic	Confirmed Syndromic	*p*-value
Length of Stay	35 (21–81)	17 (9–29)	39 (29–81)	129 (67–178)	<0.001	29 (14–42)	35 (21–78)	47 (21–104)	0.026
Disposition at Discharge					<0.001				0.401
Home	190 (87.2%)	67 (90.5%)	107 (89.9%)	16 (64.0%)		32 (94.1%)	86 (90.5%)	72 (80.9%)	
Other Acute Care Hospital	4 (1.8%)	0 (0.0%)	3 (2.5%)	1 (4.0%)		0 (0.0%)	1 (1.1%)	3 (3.4%)	
Chronic Care Facility	15 (6.9%)	0 (0.0%)	7 (5.9%)	8 (32.0%)		2 (5.9%)	4 (4.2%)	9 (10.1%)	
Expired	9 (4.1%)	7 (9.5%)	2 (1.7%)	0 (0.0%)		0 (0.0%)	4 (4.2%)	5 (5.6%)	
Weight at Discharge (grams)	4035 (3555–5275)	3620 (3180–4655)	4105 (3670–5040)	5700 (4230–7265)	<0.001	4173 (3610–4775)	3970 (3475–5035)	4290 (3560–5700)	0.555
Weight Z-score Difference^b^	−1.05 (−1.57,−0.61)	−0.93 (−1.37,−0.62)	−1.16 (−1.75,−0.65)	−0.88 (−1.80,−0.28)	0.097	−1.03 (−1.34,−0.49)	−1.02 (−1.74,−0.61)	−1.13 (−1.57,−0.65)	0.756
Failed Hearing at Discharge	90 (43.1%)	22 (32.8%)	49 (41.9%)	19 (76.0%)	0.001	7 (20.6%)	36 (39.6%)	47 (56.0%)	0.002
Age at Last Follow-Up (years)	3.7 (2.8–3.9)	3.7 (2.4–3.9)	3.6 (2.8–3.9)	3.9 (3.8–4.0)	0.002	3.5 (2.6–3.8)	3.6 (2.7–3.9)	3.9 (3.5–4.0)	0.001
Follow-Up Status after Discharge					0.013				0.456
Alive	195 (93.3%)	62 (92.5%)	113 (96.6%)	20 (80.0%)		31 (91.2%)	85 (93.4%)	79 (94.0%)	
Lost to Follow-Up	8 (3.8%)	4 (6.0%)	1 (0.9%)	3 (12.0%)		3 (8.8%)	3 (3.3%)	2 (2.4%)	
Expired	6 (2.9%)	1 (1.5%)	3 (2.6%)	2 (8.0%)		0 (0.0%)	3 (3.3%)	3 (3.6%)	
Total Mortality During Study Period	15 (6.9%)	8 (10.8%)	5 (4.2%)	2 (8.0%)	0.189	0 (0.0%)	7 (7.4%)	8 (9.0%)	0.210
Miscellaneous Procedures During Study Period									
Cleft Palate Repair	65 (29.8%)	15 (20.3%)	46 (38.7%)	4 (16.0%)	0.008	15 (44.1%)	30 (31.6%)	20 (22.5%)	0.055
Gastrostomy Tube	93 (42.7%)	20 (27.0%)	49 (41.2%)	24 (96.0%)	<0.001	5 (14.7%)	37 (38.9%)	51 (57.3%)	<0.001
Gastrostomy Tube Removal	12 (12.9%)	4 (20.0%)	7 (14.3%)	1 (4.2%)	0.248	2 (40.0%)	5 (13.5%)	5 (9.8%)	0.192
Tongue Lip Adhesion	13 (6.0%)	4 (5.4%)	7 (5.9%)	2 (8.0%)	0.839	2 (5.9%)	3 (3.2%)	8 (9.0%)	0.233
Tonsillectomy and Adenoidectomy	18 (8.3%)	4 (5.4%)	13 (10.9%)	1 (4.0%)	0.422	5 (14.7%)	7 (7.4%)	6 (6.7%)	0.338
Myringotomy and Tubes	84 (38.5%)	18 (24.3%)	55 (46.2%)	11 (44.0%)	0.007	15 (44.1%)	40 (42.1%)	29 (32.6%)	0.315
Feeding Route at Discharge					0.004^e^				0.055^e^
Exclusively by Mouth	40 (19.1%)	19 (28.4%)	21 (17.9%)	0 (0.0%)		10 (29.4%)	20 (22.0%)	10 (11.9%)	
Partial Nasogastric Tube Feeding	62 (29.7%)	19 (28.4%)	42 (35.9%)	1 (4.0%)		15 (44.1%)	25 (27.5%)	22 (26.2%)	
Nasogastric Tube Feeding Only	35 (16.7%)	16 (23.9%)	18 (15.4%)	1 (4.0%)		4 (11.8%)	17 (18.7%)	14 (16.7%)	
Gastrostomy Tube Feeding	72 (34.4%)	13 (19.4%)	36 (30.8%)	23 (92.0%)		5 (14.7%)	29 (31.9%)	38 (45.2%)	
Respiratory Status at Discharge					<0.001^f^				0.010^f^
Room Air	142 (67.9%)	47 (70.1%)	95 (81.2%)	0 (0.0%)		27 (79.4%)	68 (74.7%)	47 (56.0%)	
NC/HFNC^c^	11 (5.3%)	4 (6.0%)	7 (6.0%)	0 (0.0%)		2 (5.9%)	3 (3.3%)	6 (7.1%)	
CPAP/NIMV^d^	24 (11.5%)	16 (23.9%)	8 (6.8%)	0 (0.0%)		3 (8.8%)	11 (12.1%)	10 (11.9%)	
Invasive Ventilation	2 (1.0%)	0 (0.0%)	2 (1.7%)	0 (0.0%)		0 (0.0%)	0 (0.0%)	2 (2.4%)	
Tracheostomy	30 (14.4%)	0 (0.0%)	5 (4.3%)	25 (100.0%)		2 (5.9%)	9 (9.9%)	19 (22.6%)	
Feeding Route at Last Clinic Visit					<0.001^e^				<0.001^e^
Exclusively by Mouth	119 (59.5%)	43 (68.3%)	73 (63.5%)	3 (13.6%)		27 (87.1%)	57 (65.5%)	35 (42.7%)	
Partial Nasogastric Tube Feeding	1 (0.5%)	1 (1.6%)	0 (0.0%)	0 (0.0%)		1 (3.2%)	0 (0.0%)	0 (0.0%)	
Nasogastric Tube Feeding Only	3 (1.5%)	1 (1.6%)	2 (1.7%)	0 (0.0%)		0 (0.0%)	2 (2.3%)	1 (1.2%)	
Gastrostomy Tube Feeding	77 (38.5%)	18 (28.6%)	40 (34.8%)	19 (86.4%)		3 (9.7%)	28 (32.2%)	46 (56.1%)	
Respiratory Status at Last Clinic Visit					<0.001^f^				0.002^f^
Room Air	156 (78.0%)	56 (88.9%)	96 (83.5%)	4 (18.2%)		30 (96.8%)	70 (80.5%)	56 (68.3%)	
NC/HFNC^c^	2 (1.0%)	2 (3.2%)	0 (0.0%)	0 (0.0%)		0 (0.0%)	0 (0.0%)	2 (2.4%)	
CPAP/NIMV^d^	16 (8.0%)	5 (7.9%)	10 (8.7%)	1 (4.5%)		0 (0.0%)	10 (11.5%)	6 (7.3%)	
Tracheostomy	26 (13.0%)	0 (0.0%)	9 (7.8%)	17 (77.3%)		1 (3.2%)	7 (8.0%)	18 (22.0%)	

^a^The categorization of MDO and tracheostomy is based on the initial surgical treatment.

^b^Weight Z-score at discharge - birthweight Z-score.

^c^Standard nasal cannula or high-flow nasal cannula.

^d^Continuous positive airway pressure or non-invasive mechanical ventilation.

^e^Comparing feeding exclusively by mouth vs. all kinds of tube feedings.

^f^Comparing room air vs. all kinds of respiratory support.

# Bonferroni correction applied (*p* = 0.05/14 = 0.0036) to outcomes separately for treatment group and syndromic status.

## Bonferroni correction for feeding route and respiratoy status at admission, discharge, and follow-up were grouped together (*p* = 0.05/6 = 0.0083, separately for treatment group and syndromic status).

Only 19% of the study cohort were able to feed exclusively orally at discharge with those who underwent tracheostomy all need tube feeding and there were no significant differences between MDO and medical treatment. (Table 3 and Fig. 1B). Overall 32% of the study cohort still required some level of respiratory support at discharge, and there were no significant differences between MDO and medical treatment (Table 3 and Fig. 1E). Higher proportion of patients with genetic syndromes needed respiratory support at discharge but not significant after Bonferroni’s correction (*p* = 0.010).

The median age at last follow-up for our cohort was 3.7 years. The overall mortality during the study period was 15/218 (6.9%). The proportion of patients able to feed exclusively by mouth and in room air without respiratory support at last follow-up prior to age of four years increased to 60% and 78%, respectively, without significant differences between MDO and medical treatment (Table 3 and Fig. 1C, F). Those who underwent tracheostomy had significantly the lowest rate of feeding exclusively by mouth (14%) or in room air (18%) at last follow-up, *p* < 0.001 (Table 3 and Fig. 1C). A higher proportion of patients with confirmed syndromes required tube feeding (57%) at last follow-up compared to patients with suspected syndromes (35%) or non-syndromic patients (13%), *p* < 0.001 (Table 3 and Fig. 1C). Patients with confirmed syndromes were also more likely to still require respiratory support (32%) at last follow-up compared to patients with suspected syndromes (20%) or non-syndromic patients (3.2%), *p* = 0.002 (Table 3 and Fig. 1F). The overall study cohort during the study period underwent the following procedures (Table 3): cleft palate repair (30%), gastrostomy tube (43%), tongue lip adhesion (6.0%), tracheostomy (16%), tonsillectomy and adenoidectomy (8.3%), and myringotomy/tubes (39%).

Multivariable logistic regression analyses for feeding and respiratory outcomes at follow-up are shown in Table 4. MDO was not associated with feeding or respiratory status at follow-up compared to medical treatment. Tracheostomy was significantly associated with lower odds of feeding exclusively by mouth (aOR: 0.09 [0.02–0.39]) as well as lower odds of room air without any support (aOR: 0.03 [0.01–0.12]) at follow-up. Patients with confirmed syndromes were less likely to feed exclusively by mouth at follow-up (aOR: 0.19 [0.06–0.63]) compared to non-syndromic patients. As a secondary analysis, adding cleft repair or admission source as a covariate in these multivariable models yielded similar results.

**Table 4:** Multivariable^a^ logistic regressions for feeding route and respiratory support at last follow-up.

	PO^b^ at Follow-Up			RA^c^ at Follow-Up		
	aOR	95% CI	*p*-value	aOR	95% CI	*p*-value
Treatment Group^d^						
Medical	Reference			Reference		
MDO	0.86	0.40–1.82	0.685	0.91	0.31–2.64	0.858
Tracheostomy	0.09	0.02–0.39	0.001	0.03	0.01–0.12	<0.001
Syndromic Status						
Non-Syndromic	Reference			Reference		
Suspected Syndromic	0.46	0.14–1.53	0.207	0.26	0.03–2.29	0.224
Confirmed Syndromic	0.19	0.06–0.63	0.007	0.17	0.02–1.51	0.111

^a^Adjusted for gestational age, birthweight, sex, race/ethnicity, intubation status at birth.

^b^Feeding exclusively by mouth.

^c^Room air without any respiratory support.

^d^The categorization of MDO and tracheostomy is based on the initial surgical treatment.

## Discussion

This retrospective cohort study of 218 infants with micrognathia admitted between 1/1/2010 and 2/28/2019 for evaluation of micrognathia with median age at last follow-up of 3.7 years demonstrates a significant number of patients continued to require tube feeding and respiratory support at discharge and also throughout the first four years of life. Patients who underwent MDO during their neonatal hospitalization had significant improvement in obstructive sleep apnea following surgery. Although patients who needed MDO surgery generally had more severe obstructive sleep apnea compared to those underwent medical treatment only, after MDO they were equally likely to be in room air without respiratory support and feeding exclusively by mouth both at hospital discharge and at follow-up. Not surprisingly, the majority of those who required tracheostomy still needed tube feeding and respiratory support even at last follow-up close to four years of age. A large proportion of infants with micrognathia required gastrostomy (43%) and myringotomy/tubes (39%) during the study period. These findings suggest that infants with micrognathia require multidisciplinary support after neonatal hospitalization and patients selected to undergo MDO have favorable outcomes similar to those without surgical treatment despite suffering from more airway obstruction initially.

In our cohort, 81% of infants with micrognathia needed tube feeding at discharge and 41% at follow-up up to four years of age continued to require tube feeding. Overall, 43% of patients had gastrostomy placement. Similar to prior studies, syndromic patients were less likely to exclusively feed by mouth at discharge and at follow-up, and more likely to have gastrostomy [7, 10]. The exact proportion of patients requiring feeding tubes differs slightly likely due to patient selection and variation of practice from different institutions [13]. We found that infants with micrognathia had poor weight gain during their neonatal hospitalization, which was more pronounced in those undergoing MDO, similar to Dorise et al.’s study [20]. Other studies have shown that infants with micrognathia have initial poor weight gain and then improved weight gain following MDO [21–24], but may take 1-3 years for catch-up growth [11, 25]. Unfortunately, the longitudinal weight trajectory after discharge was not recorded in our study. These findings suggest that infants with micrognathia are at risk for poor growth. A large proportion of patients in our cohort required tube feedings following discharge, therefore attention to nutrition early as well as close outpatient follow-up for continued feeding therapy and nutrition monitoring is important for optimal growth.

Our patients who underwent MDO had significant improvement in polysomnography parameters including OAHI and SpO_2_ nadir, confirming the findings from prior studies in this larger cohort [12, 14]. Similar to prior studies [9, 26], we also showed infants with micrognathia are at risk of hearing loss (43% failed hearing at discharge), emphasizing the importance of audiology evaluation in infants with micrognathia and subsequent follow-up.

Management of patients with micrognathia at our institution involves multidisciplinary evaluation and treatment. We had formalized a protocol for general guidance and standardization of care that was launched circa 2017 (*n* = 78) [18], and the overall management of patients prior to 2017 also followed similar conceptual framework (*n* = 140). Our management decisions are similar to published treatment algorithms from other centers [27–30], with the exception that tongue-lip adhesions are not routinely considered at our center. Additional quality improvement studies evaluating protocol compliance and outcomes are warranted to provide optimal care.

There are several strengths and important implications from our study. Our cohort is one of the largest (218 patients) whereas the majority of prior studies on micrognathia have small number of patients mostly fewer than 50. Our cohort was from the recent decade with the majority of the operative group undergoing MDO, which is consistent with the current trend [6] thereby providing more updated information regarding current practice. Furthermore, we also followed our patients up to four years of age in this study allowing the assessment of long-term feeding and respiratory outcomes and the need for additional procedures. Our study suggests infants with micrognathia with or without surgical intervention require long-term multidisciplinary support as many of these patients still require tube feeding and respiratory support years after discharge.

Our study has some limitations. Our retrospective study is subject to selection bias where patients were evaluated and selected to undergo MDO vs. tracheostomy based on multidisciplinary expertise. We attempted to adjust the bias in multivariable logistic regressions to include baseline characteristics and syndromic status, however, our comparative findings should be viewed as associations rather than causal relationships. Nevertheless, the favorable outcomes after MDO in our study are consistent with prior studies. Future studies should focus on identifying ideal candidates for MDO and optimizing postoperative care such as pain management, timing of extubation, feeding and respiratory support, and multidisciplinary follow-up to further improve outcomes. Although our study is one of the largest single-center cohort of micrognathia, the sample size may not be powered enough for the multiple outcomes studied especially after adjustment for multiple comparisons. However, our results will provide insights for the design of future studies. There are many genetic syndromes associated with micrognathia as was observed in our study [2, 3], and sub-analysis of specific syndrome was not possible with the size of this single-center cohort, but should be the focus of future multi-center studies. The long-term impact of micrognathia and surgical intervention on dental [31, 32], speech [33–37], or neurodevelopmental outcomes [38, 39], was not captured in this retrospective cohort, but are important outcomes to be included in future clinical trials.

In conclusion, patients who underwent tracheostomy were associated with tube feeding and respiratory support at follow-up and patients who underwent MDO had similar favorable long-term feeding and respiratory outcomes compared to those needing only medical treatment. A large proportion of infants with micrognathia still required feeding assistance and/or respiratory support at follow-up up to four years. Improved understanding of potential trajectories of feeding and respiratory outcomes provided by this study can facilitate planning for multidisciplinary care and family counseling, with referral to appropriate support services.

## Supplementary information

Supplemental Table 1: List of Genetic Syndromes

Supplemental Table 2: Polysomnography (PSG) Results

## Data Availability

The datasets generated and/or analyzed during the current study are available from the corresponding author on reasonable request.
